# Aerobic training reduces oxidative stress in skeletal muscle of rats exposed to air pollution and supplemented with chromium picolinate

**DOI:** 10.1080/13510002.2018.1475993

**Published:** 2018-05-18

**Authors:** Bruna Marmett, Ramiro Barcos Nunes, Kellen Sábio de Souza, Pedro Dal Lago, Cláudia Ramos Rhoden

**Affiliations:** a Laboratory of Atmospheric Pollution, Graduate Program in Health Science, Federal University of Health Sciences of Porto Alegre (UFCSPA), Porto Alegre, Brazil; b Research Department, Sul-Rio-Grandense Federal Institute of Education, Science and Technology, Gravataí, Brazil; c Laboratory of Experimental Physiology, Federal University of Health Sciences of Porto Alegre (UFCSPA), Porto Alegre, Brazil

**Keywords:** Air pollution, chromium tripicolinate, dietary supplements, exercise, oxidative stress

## Abstract

**Objective:** The purpose of this study was to investigate the effects of chromium picolinate (CrPic) supplementation associated with aerobic exercise using measures of oxidative stress in rats exposed to air pollution.

**Methods:** Sixty-one male Wistar rats were divided into eight groups: residual oil fly ash (ROFA) exposure and sedentary (ROFA-SED); ROFA exposure, sedentary and supplemented (ROFA-SED-CrPic); ROFA exposure and trained (ROFA-AT); ROFA exposure, supplemented and trained (ROFA-AT-CrPic); sedentary (Sal-SED); sedentary and supplemented (Sal-SED-CrPic); trained (Sal-AT); and supplemented and trained (Sal-AT-CrPic). Rats exposed to ROFA (air pollution) received 50 µg of ROFA daily via intranasal instillation. Supplemented rats received CrPic (1 mg/kg/day) daily by oral gavage. Exercise training was performed on a rat treadmill (5×/week). Oxidative parameters were evaluated at the end of protocols.

**Results:** Trained groups demonstrated lower gain of body mass (*P *< .001) and increased exercise tolerance (*P *< .0001). In the gastrocnemius, trained groups demonstrated increased SOD activity (*P *< .0001) and decrease levels of TBARS (*P *= .0014), although CAT activity did not differ among groups (*P *= .4487).

**Conclusion:** Air pollution exposure did not lead to alterations in oxidative markers in lungs and heart, and exercise training was responsible for decreasing oxidative stress of the gastrocnemius.

## 1. Introduction

Air pollution is currently classified as a leading environmental cause of cancer and ranked as one of the top 10 causes of disability [1–3]. Furthermore, polluted air is related to premature mortality, estimated to cause a global mortality burden of more than 3 million premature deaths/year. The projections of emission scenarios indicate that these values could double by 2050, if any measure of air quality control could be made [4,5]. There is strong evidence that pollutants present in air are responsible for the detrimental effects of air pollution, triggering oxidative stress and systemic inflammation [3,6,7]. These effects contribute to the pathological mechanism, increasing the susceptibility of the population to developing chronic diseases [6,7].

Physical inactivity is one of the most significant public health problems of the twenty-first century and is the fourth leading cause of death worldwide [8,9], whereas physical activity demonstrates well-established health benefits [10–12]. Exercise training induces the formation of reactive oxygen species (ROS), which act as important mediators of physiologic signaling and cellular adaptations, modulation of muscle contraction, regulation of antioxidant protection and repair of oxidative damage [13,14]. The majority of studies investigating air pollution and exercise, however, have demonstrated controversial findings [13–16].

Chromium picolinate (CrPic) is a trivalent chromium complex largely used to control glucose levels and improve insulin sensitivity. Furthermore, CrPic supplementation has exhibited antioxidant activity in animals with established oxidative stress, being responsible for increasing glutathione (GSH) levels, catalase (CAT) and superoxide dismutase (SOD) activity, in addition to decreasing lipid peroxidation [17–22]. CrPic antioxidant activity has not been investigated in association with air pollution exposure, however. Considering the antioxidant properties of this supplement, it could possibly decrease the damaging effects of air pollution on health, attenuating the action of oxidative molecules and, subsequently, the damage they induce [23,24].

The aim of the present study was to examine the effects of CrPic supplementation associated with aerobic exercise during subchronic air pollution exposure in measures of oxidative stress. In addition, we investigated the adaptation of aerobic exercise in groups exposed to air pollution and supplemented with CrPic.

We tested the hypothesis that air pollution exposure may increase body mass and decrease exercise tolerance, as well as inducing oxidative damage in the lungs, heart and gastrocnemius, whereas supplementation of CrPic and aerobic training protocols may lead to beneficial effects on those variables.

## 2. Materials and methods

### 2.1. Animals

This study was performed using 61 male Wistar rats (45 days old) obtained from the Animal Breeding Unit of the Universidade Federal de Ciências da Saúde de Porto Alegre (UFCSPA). The animals were housed under standard conditions (food and water *ad libitum*, temperature between 22 and 24°C, light–dark cycle of 12 h). The handling of the animals obeyed resolutions of the National Council on Animal Experimentation and all procedures were in accordance with the Guide for the Care and Use of Laboratory Animals adopted by the National Institute of Health (NIH-USA). This study was approved by CEUA/UFCSPA, under the protocol number 159/15.

## 2.2. Experimental design

Sixty-one male Wistar rats were divided into eight experimental groups: residual oil fly ash (ROFA) exposure and sedentary (ROFA-SED, *n* = 8); ROFA exposure, sedentary and supplemented (ROFA-SED-CrPic, *n* = 6); ROFA exposure and trained (ROFA-AT, *n* = 8); ROFA exposure, supplemented and trained (ROFA-AT-CrPic, *n* = 7); sedentary (Sal-SED, *n* = 8); sedentary and supplemented (Sal-SED-CrPic, *n* = 8); trained (Sal-AT, *n* = 8); supplemented and trained (Sal-AT-CrPic, *n* = 8). Intranasal instillation of ROFA and CrPic supplementation protocols were performed daily for 90 days, and the training protocol was performed for the same period of time (5×/week).

### 2.3. Intranasal instillation of ROFA

Animals exposed to air pollution received 50 µg of ROFA via intranasal instillation daily for 90 days. ROFA was applied as a recognized form of particulate matter. The dose used represents a concentration of 29 µg/m³, which is the value found in a polluted city [25]. ROFA particles were collected from an electrostatic precipitator installed in one of the chimneys of a large steel plant in São Paulo, Brazil. Characterization of ROFA is included in Table 1. A suspension of 50 µg of ROFA was prepared in 10 µl of sterile saline solution. When rats were 60 and 90 days old, ROFA suspensions of 50 µg were prepared in 20 and 30 µl sterile saline solutions, respectively. The volume was adjusted to ensure that the suspension would reach the lungs, considering that as the rats’ respiratory systems developed, a greater volume would be necessary [26,27]. Control groups underwent the same instillation protocol, but received only saline.

**Table 1.** Characterization of metals in residual oil fly ash.

Metal	µg/g (mean ± SD)
Pb	3.1 ±** **0.09
Al	789.9 ±** **23.28
Zn	20.3 ± 0.04
Cd	0.04 ±** **0.002
Ba	30.2 ± 0.31
Cu	9.7 ±** **0.15
Ni	287.0 ±** **10.8
As	4.1 ±** **0.05
Se	7.5 ± 0.20
Mn	48.3 ±** **0.98
Sr	8.4 ±** **0.16
Sb	2.3 ±** **0.57
Fe	20,397.2 ± 283.3
Mg	372.5 ±** **1.93
P	388.5 ±** **255.8
Cr	7.6 ±** **0.23

Note: Pb: Lead; Al: Aluminum; Zn: Zinc; Cd: Cadmium; Ba: Barium; Cu: Copper; Ni: Nickel; As: Arsenic; Se: Selenium; Mn: Manganese; Sr: Strontium; Sb: Antimony; Fe: Iron; Mg: Magnesium; P: Phosphorus; and Cr: Chromium.

### 2.4. Chromium picolinate supplementation

Supplemented groups received 1 mg/kg of CrPic in 1 ml sterile saline solution (presentation form: powder, with purity of ≥ 98%, Pharma Nostra^®^, Brazil) by oral gavage daily for 90 days. Animals were weighed every 15 days to allow for dose adjustment [19]. Control groups underwent the same supplementation protocol, but received only saline.

### 2.5. Exercise tolerance test

All rats underwent an exercise tolerance test to measure their maximal running capacity before and after the period of experiments. First, animals were subjected to an adaptation period of five days and run for 10 min/day [28]. The test consisted of running on an electric treadmill with an inclination of 15°, starting with a speed of 5 m/min and increasing by an increment of 5 m/min every 3 min until exhaustion. Exhaustion was established as the time at which the animal was unable to run for at least 15 s, even while receiving an electrical stimulus (1.5 μA) [29].

### 2.6. Training protocol

The animals in trained groups underwent aerobic exercise training, which was performed on a motorized treadmill five days/week with a moderate intensity of 70% for 90 days. The running time started at 20 min on the first week and was extended by 10 min/week until 50 min/day was reached (all rats were running) [29,30].

### 2.7. Tissue collection

After 90 days of experimental protocols, animals were anesthetized via exposure to isoflurane in oxygen (induction 5%, 2 L/min) for 5 min in an induction chamber and then euthanized via the exsanguination method. Lungs, heart and gastrocnemius were dissected and stored in −80°C for subsequent analyses of oxidative stress.

### 2.8. Oxidative stress analysis

#### 2.8.1. Tissue preparation

To prepare tissues, lungs, heart and gastrocnemius were defrosted, weighed in an analytical balance and homogenized in KPi buffer (KCl 1.15%, pH 7.4) containing protein inhibitors. Homogenization was performed in a tissue homogenizer (CT-136.1, Cientec^®^), after which samples were centrifuged and supernatants were stored at −80°C until oxidative stress analyses were conducted.

#### 2.8.2. Protein concentration

Protein concentration of the tissues homogenates was measured via Bradford’s method [31] using bovine serum albumin as a standard. The sample absorbance was determined at 595 nm, using a Lambda 35 spectrophotometer (Perkin-Elmer of Brazil, SP, Brazil).

#### 2.8.3. Superoxide dismutase activity

SOD activity was determined based on the inhibition of pyrogallol auto-oxidation by the enzyme, following the method described by Marklund and Marklund [32]. Sample absorbances were determined using a Lambda 35 spectrophotometer (Perkin-Elmer of Brazil, SP, Brazil), at 420 nm after 60 and 120 s. The results were expressed as USOD/mg of total protein.

#### 2.8.4. CAT activity

CAT activity was determined based on the decomposition of hydrogen peroxide at 25°C, following the method described by Aebi [33]. Sample absorbances were determined using a Lambda 35 spectrophotometer (Perkin-Elmer of Brazil, SP, Brazil), at 240 nm for 120 s. The results were expressed in nmol/mg of total protein.

#### 2.8.5. Thiobarbituric acid-reactive substances

To determine lipid peroxidation, thiobarbituric acid-reactive substances (TBARS) levels were measured according to the technique described by Esterbauer and Cheeseman [34]. Sample absorbances were determined at 535 nm using a Lambda 35 spectrophotometer (Perkin-Elmer of Brazil, SP, Brazil). TBARS concentration was expressed in nmol/mg of total proteins. To calculate TBARS levels, a standard curve generated based on known concentrations of 100 nmol/ml 1,1,3,3-tetrametoxypropane in 1% H_2_SO_4_ solution was utilized.

### 2.9. Statistical analysis

Data are expressed as mean ± standard deviation (SD). Statistical analyses were begun using the Kolmogorov–Smirnov test to evaluate normality of all variables. Two-way repeated measures analysis of variance (ANOVA) was then performed, followed by Tukey’s *post hoc* test to compare body mass variables between treatment and control groups. Other variables were analyzed using one-way ANOVA, followed by Tukey’s *post hoc* test to compare between treatment and control groups. For statistical analysis and graphics creation, SigmaPlot version 12.0 for Windows (Systat Software, Inc.) and GraphPad Prism version 6.0 for Windows (Prism 6; GraphPad Software, Inc.) were used. A *P* <  .05 was considered statistically significant.

## 3. Results

**Figure 1.**
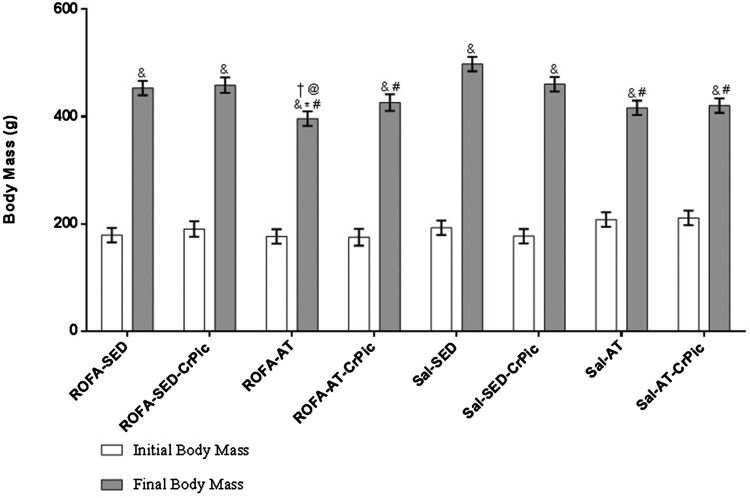
Body mass before and after 12 weeks of chromium picolinate (CrPic) supplementation and aerobic exercise intervention in rats exposed to residual oil fly ash (ROFA). Values presented as mean ± SD. Statistical analysis: two-way repeated measures analysis of variance (ANOVA) followed by Tukey’s *post hoc* test. ROFA-SED, ROFA exposure and sedentary (*n* = 8); ROFA-SED-CrPic, ROFA exposure, sedentary and supplemented (*n* = 6); ROFA-AT, ROFA exposure and trained (*n* = 8); ROFA-AT-CrPic, ROFA exposure, supplemented and trained (*n* = 7); Sal-SED, sedentary (*n* = 8); Sal-SED-CrPic, sedentary and supplemented (*n* = 8); Sal-AT, trained (*n* = 8); Sal-AT-CrPic, supplemented and trained (*n* = 8). Symbols represent comparisons among groups based on the *post hoc* analysis: &*P* < .05 vs. Initial Body Mass; *P < .05 vs. ROFA-SED; @*P* < .05 vs. ROFA-SED-CrPic; #*P* < .05 vs. Sal-SED; †*P* < .05 vs. Sal-SED-CrPic.

Our study started with 64 animals; however, during the study, there were three losses (one from the ROFA-AT-CrPic group and two from the ROFA-SED-CrPic group) due to causes not related to the experiments (data not shown). Initial body mass did not differ among groups (*P* > .05) and at the end of the study, all groups showed a mean increase in body mass of 236% (*P* < .001). Furthermore, when final body mass was compared among groups, the trained groups demonstrated lower gain of body mass in comparison to the Sal-SED group (*P < *.05; Figure 1).

**Figure 2.**
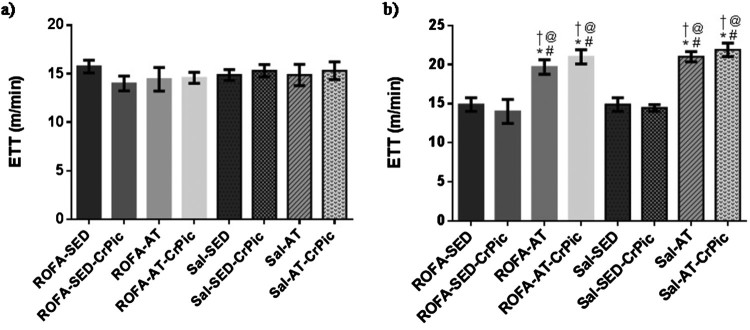
Exercise tolerance test before and after 12 weeks of chromium picolinate (CrPic) supplementation and aerobic exercise intervention in rats exposed to residual oil fly ash (ROFA). (a) Initial exercise tolerance test; (b) final time of exercise tolerance test. Values presented as mean ± SD. Statistical analysis: one-way ANOVA followed by Tukey’s *post hoc* test. ROFA-SED, ROFA exposure and sedentary (*n* = 8); ROFA-SED-CrPic, ROFA exposure, sedentary and supplemented (*n* = 6); ROFA-AT, ROFA exposure and trained (*n* = 8); ROFA-AT-CrPic, ROFA exposure, supplemented and trained (*n* = 7); Sal-SED, sedentary (*n* = 8); Sal-SED-CrPic, sedentary and supplemented (*n* = 8); Sal-AT, trained (*n* = 8); Sal-AT-CrPic, supplemented and trained (*n* = 8). Symbols represent comparisons among groups based on the *post hoc* analysis: *P < .05 vs. ROFA-SED; @ *P *< .05 vs. ROFA-SED-CrPic; # *P *< .05 vs. Sal-SED; †*P* < .05 vs. Sal-SED-CrPic.

Exercise tolerance tests performed at the beginning of the study demonstrated no difference among groups (*P *= .8879). In the final exercise tolerance test, however, an increase in exercise tolerance was observed in the trained groups when compared to the sedentary groups (*P *< .0001; Figure 2).

**Figure 3.**
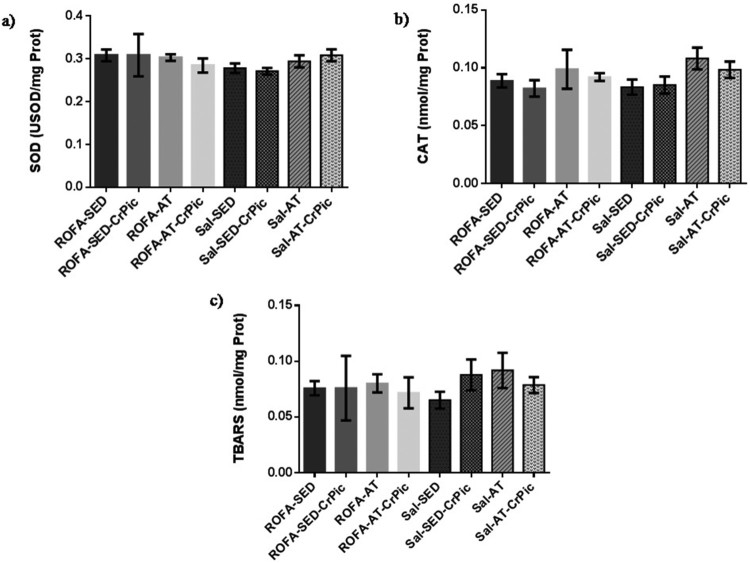
Oxidative stress in lung tissue after 12 weeks of chromium picolinate (CrPic) supplementation and aerobic exercise intervention in rats exposed to residual oil fly ash (ROFA). Analyses of (a) superoxide dismutase (SOD) activity in lung tissue; (b) catalase (CAT) activity in lung tissue and (c) thiobarbituric acid-reactive substance (TBARS) levels in lung tissue. Values presented as mean ± SD. Statistical analysis: one-way ANOVA followed by Tukey’s *post hoc* test. ROFA-SED, ROFA exposure and sedentary (*n* = 8); ROFA-SED-CrPic, ROFA exposure, sedentary and supplemented (*n* = 6); ROFA-AT, ROFA exposure and trained (*n* = 8); ROFA-AT-CrPic, ROFA exposure, supplemented and trained (*n* = 7); Sal-SED, sedentary (*n* = 8); Sal-SED-CrPic, sedentary and supplemented (*n* = 8); Sal-AT, trained (*n* = 8); Sal-AT-CrPic, supplemented and trained (*n* = 8).

**Figure 4.**
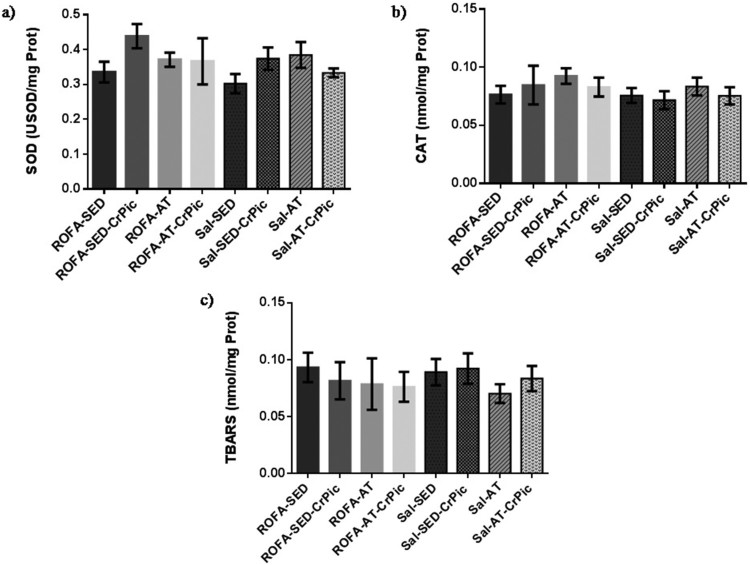
Oxidative stress in heart tissue after 12 weeks of chromium picolinate (CrPic) supplementation and aerobic exercise intervention in rats exposed to residual fly oil ash (ROFA). Analyses of (a) superoxide dismutase (SOD) activity in heart tissue; (b) catalase (CAT) activity in heart tissue and (c) thiobarbituric acid-reactive substance (TBARS) levels in heart tissue. Values presented as mean ± SD. Statistical analysis: one-way ANOVA followed by Tukey’s *post hoc* test. ROFA-SED, ROFA exposure and sedentary (*n* = 8); ROFA-SED-CrPic, ROFA exposure, sedentary and supplemented (*n* = 6); ROFA-AT, ROFA exposure and trained (*n* = 8); ROFA-AT-CrPic, ROFA exposure, supplemented and trained (*n* = 7); Sal-SED, sedentary (*n* = 8); Sal-SED-CrPic, sedentary and supplemented (*n* = 8); Sal-AT, trained (*n* = 8); Sal-AT-CrPic, supplemented and trained (*n* = 8).

Activity levels of SOD and CAT in lung tissue did not differ among groups (*P *= .2756 and *P *= .1198, respectively), nor did TBARS levels differ among groups (*P *= .7189; Figure 3). Similarly, in heart tissue, no differences were observed among groups in relation to SOD (*P *= .0763) and CAT (*P *= .4999) activity levels, or TBARS levels (*P = *.8656; Figure 4).

**Figure 5.**
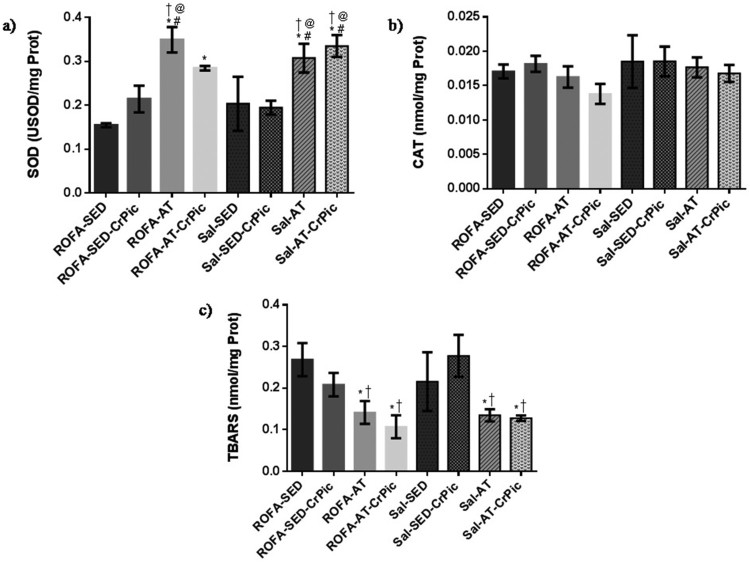
Oxidative stress in gastrocnemius muscle after 12 weeks of chromium picolinate (CrPic) supplementation and aerobic exercise intervention in rats exposed to residual oil fly ash (ROFA). Analyses of (a) superoxide dismutase (SOD) activity in gastrocnemius tissue; (b) catalase (CAT) activity in gastrocnemius tissue and (c) thiobarbituric acid-reactive substance (TBARS) levels in gastrocnemius tissue. Values presented as mean ± SD. Statistical analysis: one-way ANOVA followed by Tukey’s *post hoc* test. ROFA-SED, ROFA exposure and sedentary (*n* = 8); ROFA-SED-CrPic, ROFA exposure, sedentary and supplemented (*n* = 6); ROFA-AT, ROFA exposure and trained (*n* = 8); ROFA-AT-CrPic, ROFA exposure, supplemented and trained (*n* = 7); Sal-SED, sedentary (*n* = 8); Sal-SED-CrPic, sedentary and supplemented (*n* = 8); Sal-AT, trained (*n* = 8); Sal-AT-CrPic, supplemented and trained (*n* = 8). Symbols represent comparisons among groups based on the *post hoc* analysis: *P < .05 vs. ROFA-SED; @*P *< .05 vs. ROFA-SED-CrPic; #*P *< .05 vs. Sal-SED; †*P *< .05 vs. Sal-SED-CrPic.

In the gastrocnemius, SOD activity was higher in the groups that underwent aerobic training (ROFA-AT, Sal-AT and Sal-AT-CrPic) than in all sedentary groups (ROFA-SED, ROFA-SED-CrPic, Sal-SED and Sal-SED-CrPic; *P *< .0001; Figure 5). No differences in CAT activity were observed among groups (*P *= .4487), but levels of TBARS were lower in trained groups when compared to the ROFA + SED and Sal-SED-CrPic groups (*P *= .0014).

## 4. Discussion

The main findings of the present study were an improvement in exercise tolerance and a reduction in oxidative stress in the gastrocnemius muscle in rats that underwent aerobic training. These findings were evidenced by an increase in both exercise tolerance and SOD activity, and a decrease in TBARS levels in the gastrocnemius muscle. Oxidative stress parameters evaluated in lung and heart tissues, however, did not differ among groups.

Regarding body mass, all groups exhibited increases in body mass during the study, which was expected due to the growth process. Conversely, aerobic exercise could attenuate mass gain, likely due to increased energy expenditure, resulting in a negative energy balance and, consequently, a reduction of mass gain. Such an outcome was observed in an experimental study by Cigarroa et al. [35], which demonstrated that a treadmill intervention could counterbalance the mass gain of animals fed a cafeteria diet.

Our findings suggest that aerobic training leads to exercise tolerance, evidenced by an increased maximal velocity, as demonstrated in experimental studies that use aerobic training [28,29]. The repetitive muscle contraction during exercise training can lead to a variety of responses that increase aerobic metabolism capacity and exercise tolerance [36–38].

In the present study, no differences in SOD and CAT activity, or TBARS levels, in the lungs and heart were observed among groups. Consistent with our results, a study reporting exposure to 50 and 250 µg of ROFA for 90 days also observed no change in oxidative stress markers, such as SOD, CAT and TBARS [39]. Interestingly, acute inhalation of concentrated ambient particles and ROFA has been found to lead to potential oxidant injuries, followed by an up-regulation of antioxidant defenses. After a 24 h inhalation period, reversibility of oxidative stress occurs, suggesting that oxidants mediated by pollution exposure may trigger adaptive responses in the lungs and heart [40–43]. Moreover, our study was performed using healthy animals. In contrast, studies using different experimental models of diseases have demonstrated that an underlying condition represents the greatest risk after ROFA exposure, as once oxidants and inflammatory migration occur at sites where there is a pre-established inflammation; this is not observed in healthy animals [44–46].

Regarding evaluations of oxidative stress in the gastrocnemius, we found increased SOD activity together with decreased TBARS levels in trained groups, suggesting a positive effect of aerobic exercise on oxidative stress in the gastrocnemius muscle. Supporting our results, muscle activity during exercise increases ROS formation and simultaneously promotes an increase in the antioxidant defense system, as well as improving resistance to oxidative stress [38,47]. Furthermore, higher levels of SOD activity in skeletal muscle are related to intensity and duration of exercise, as evidenced by studies reporting that higher intensities and longer durations of exercise were associated with increased SOD activity [47].

We found no significant difference in CAT activity among groups, indicating no alteration in levels of this enzyme in skeletal muscle in response to exercise. Likely, CAT activity did not exhibit changes due to the action of other antioxidant mechanisms in skeletal muscle that neutralize hydrogen peroxide, such as glutathione peroxidase (GPx) and peroxiredoxins. Endurance exercise promotes an increase of 20–177% in GPx activity and peroxiredoxins are constitutively secreted from the skeletal muscle, becoming more abundant than CAT and GPx [47–49].

Notably, the reduced TBARS levels observed in trained groups in our study could be associated with the increased SOD activity, which may prevent against lipid peroxidation, considering that ROS generation induced by exercise is a stimulus for activating the expression of antioxidant enzymes [6,50]. Naturally, a decrease in TBARS levels would be a consequence of the SOD activity [38,47].

In relation to the lack of effect of supplementation, CrPic can play an antioxidant effect when there is an oxidative disruption, because CrPic supplementation could preserve the antioxidant status when there are a depletion of antioxidant enzymes and an increase in oxidative stress [51–53]. In the present study, positive action of CrPic was not observed, likely because there was no depletion of antioxidant enzymes or increased oxidative stress in our sample. Other studies investigating the effects of antioxidant supplementation have reported that supplementation has led to beneficial effects and reduced oxidative stress only in individuals with low baseline antioxidant profiles [43,51,54,55].

In conclusion, this study showed that in a healthy sample, subchronic ROFA exposure did not lead to alterations in oxidative markers. Furthermore, exercise training could decrease body mass gain and increase exercise tolerance, as well as increasing SOD activity and decreasing lipid peroxidation of skeletal muscles, such as the gastrocnemius.
